# Dietary exposure to pesticide residues from foods of plant origin and drinks in Lebanon

**DOI:** 10.1007/s10661-016-5505-y

**Published:** 2016-07-27

**Authors:** Lara Nasreddine, Maria Rehaime, Zeina Kassaify, Roula Rechmany, Farouk Jaber

**Affiliations:** 1 Department of Nutrition and Food Sciences, Faculty of Agricultural and Food Sciences, American University of Beirut, P.O.Box: 110236, Beirut, Riad El-Solh 1107 2020 Lebanon; 2 Lebanese Atomic Energy Commission—CNRS, National Council for Scientific Research (CNRS), P.O. Box 11- 8281, Beirut, Riad El Solh 107 2260 Lebanon; 3 Laboratory of Analysis of Organic Compounds (509), Faculty of Sciences I, Lebanese University, Beirut, Lebanon

**Keywords:** Adults, Dietary exposure, Lebanon, Pesticide residues, Risk characterization, Total diet study

## Abstract

This study assesses the dietary exposure of Lebanese adults to 47 pesticide residues from both foods of plant origin and drinks. The study was conducted using the Total Diet Study protocol in two different areas of Lebanon: Greater Beirut (urban) and Keserwan (semi-rural). A total of 1860 individual foods were collected, prepared, and cooked prior to analysis. Composite samples of similar foods were analyzed, following the QuEChERS Multiresidue method. Eighteen residues were detected/quantified on at least one composite sample, with 66.7 % of the results being quantifiable and 33.3 % detectable. Quantifiable levels ranged between 10.3 and 208 μg/kg. For the composite samples where residues were detected, 55 % had one residue, while 45 % had 2–4 residues. The most frequently detected/quantified pesticide residues included Chlorpyrifos, Procymidone, Primiphos methyl, Dimethoate, and Dieldrin. The dietary exposure assessment was conducted using the deterministic approach with two scenarios: (1) the lower bound (LB) approach and (2) the upper bound (UB) approach. Using the LB approach, mean estimated daily exposures were far below the acceptable daily intakes (ADIs) for all investigated residues. Using the UB approach, which tends to overestimate exposure, mean estimated daily exposures were below the ADIs for all residues except for Dieldrin (semi-rural: 128.7 % ADI; urban: 100.7 % ADI). Estimates of mean exposure to Diazinon reached 50.3 % of ADI in the urban diet and 61.9 % in the semi-rural diet. Findings of this study identify specific pesticide residues as monitoring priorities for which more comprehensive and sensitive analyses are needed in order to refine exposure assessment.

## Introduction

Chronic dietary exposure to unsafe levels of chemicals and nutritional imbalances is known to be associated with a wide array of human health disorders such as organ dysfunction and the promotion of certain types of cancer (GEMS/Food 2005). Therefore, protecting diets from chemical contaminants and nutritional inadequacies should be considered as one of the essential and priority public health functions of any country. In this context, the Global Environment Monitoring System-Food Contamination and Assessment Program (GEMS/Food 2005) of the World Health Organization (WHO) has included pesticide residues among the priority food contaminants that should be examined in each country (WHO 2002).

Pesticides are among the mostly utilized chemicals in the world, mainly for their ability to fight pests; control unwanted insects, mites, fungi, and rodents; and prevent food-borne and vector-borne diseases (Ferrer 2003; FSANZ 2002). Although the pesticides’ toxic effects are targeted toward specific pest species, the potential for adverse health effects on humans and other non-targeted species has been flagged as a public health issue (Weiss et al. 2004). Negative health effects of pesticides have been investigated in various epidemiological studies, most of them focusing on farmers’ populations and occupational exposure to high doses (Mansour 2004; FAO/WHO 2007; Nougadère et al. 2012; INSERM 2013). These studies have suggested that long-term exposure to pesticides may be associated with a broad spectrum of potential adverse health effects on humans such as carcinogenesis, neurotoxicity, cytogenetic damage, and endocrine disruption in addition to developmental, reproductive, and immunological effects (Mansour 2004; FAO/WHO 2007; Nougadère et al. 2012). For the general population, the diet is considered as the major route of exposure to pesticide residues (Lu et al. 2006; Luo and Zhang 2009; Panuwet et al. 2009; Cao et al. 2011), thus highlighting the need for rigorous investigations of the consumer’s risk associated with these residues. This is the purpose of risk assessment, which is a scientifically based process that could be subdivided into four steps: (1) *hazard identification*, (2) *hazard characterization*, (3) *exposure assessment*, and (4) *risk characterization* (Renwick 2002). *Hazard identification* is the process by which a particular chemical is causally linked to a specific negative health effect (Verger 2013). For pesticide residues, hazard identification involves a series of in vitro and in vivo studies to define the biological properties of the chemical that could lead to adverse effects (Nasreddine and Parent-Massin 2002; Renwick 2002). *Hazard characterization* consists of defining the dose–response relationship and determining for each chemical under consideration, the threshold below which the health risk is deemed negligible (Verger 2013). Identifying this threshold leads to the establishment of the acceptable daily intake (ADI), which is defined as the amount of a specific chemical that can be ingested every day for a whole human lifetime without appreciable health risks (Verger 2013). The ADI is usually generated from the lowest no-observed-adverse-effect-level (NOAEL) in the most sensitive species, using a 100-fold safety factor (Nasreddine and Parent-Massin 2002). In addition to the setting of ADIs, hazard characterization for pesticide residues leads to the identification of the acute reference dose (ARfD) and the acceptable operator exposure level (AOEL) (Renwick 2002). The ARfD is an estimate of the amount of a chemical in food and/or drinking water that can be ingested in a period of 24 h or less, without appreciable health risk to the consumer (WHO 2015). As for the AOEL, it is defined as the level of daily exposure that would not cause any adverse effect in operators who work with a particular pesticide regularly over days, weeks, or months (Renwick 2002).

*Dietary exposure assessment* consists of associating food consumption data with concentration/contamination data. It typically includes the application of statistical adjustment factors that allow conclusions about the amount of a substance being consumed on a “usual” basis or over a lifetime (FAO/WHO 2006). The fourth and final step of the risk assessment process is *risk characterization*, which consists of comparing exposure estimates with health-based guidance values such as the ADI, thus evaluating the potential health risk for the individual (Nasreddine and Parent-Massin 2002).

In Lebanon, an Eastern Mediterranean country with a population estimated at about four million, the consumer’s risk associated with exposure to pesticide residues has not been previously assessed. Only few sporadic studies have investigated the contamination of selected fruits and vegetables by pesticide residues, sometimes reporting high levels of contamination (Kawar and Dagher 1976; Boxter and Saliba 1996; Dagher et al. 1999). The monitoring of food safety by governmental bodies has also lagged behind, whereby national safety evaluation and monitoring programs of pesticide residues in food, the enforcement of regulatory measures, and the available national data on food contamination levels are particularly scarce. Even though the Lebanese Ministry of Agriculture has put in place a pesticides’ registration system whereby only the registered pesticides are legally allowed to be imported into the country, banned pesticides may still be illegally entering Lebanon due the absence of effective law enforcement mechanisms. In addition, at the farmer level, the purchase or use of pesticides is not subjected to any authorization system. Although the Lebanese Standards Institution (LIBNOR Lebanese Standards Institution 2003) has set specific MRLs to food items (LIBNOR Lebanese Standards Institution 2003), surveillance measures ensuring conformity with crop-specific maximum residue levels (MRLs) are lagging behind in the country.

In response to the need for data that contribute toward characterizing the risk for the Lebanese consumer, the present study was conducted with the aim of assessing the dietary exposure of Lebanese adults to pesticide residues from both foods of plant origin and drinks. The selection of these food items is explained by the fact that the analytical techniques and expertise available in Lebanon allow the determination of pesticide residues in low-fat food matrices. In the absence of food consumption data at the national level, this study was conducted in the Greater Beirut area, the capital and the melting pot of the country, which encompasses approximately 40 % of the Lebanese population and receives agricultural produce from all over the country as well as from abroad (Nasreddine et al. 2006). In order to provide a preliminary assessment of potential regional differences in dietary exposure levels, the study was also conducted in Keserwan, a semi-rural area situated in the Mount Lebanon Governorate, to the northeast of the capital Beirut. This region is characterized by its local production of agricultural produce (mainly fruits and vegetables), in addition to receiving food products from other areas in the country. Both study areas, Greater Beirut (urban) and Keserwan (semi-rural), were shown to significantly differ in terms of food consumption patterns, with a significantly higher intake of fruits, vegetables, pulses, nuts, cereals, and cereal-based products observed in the semi-rural area compared to the urban one (Reshmany 2010). In order to assess the dietary exposure to pesticide residues, the methodology adopted in the study is based on the total diet study (TDS) approach, also known as the market basket (MB) study (WHO INFOSAN 2006), which allows to measure average exposure levels to food chemicals and aims at evaluating the chronic public health risks associated with exposure to these chemicals. Methodologically, TDSs consist of purchasing, at the retail level, foods commonly consumed by the population or by a specific population group, processing them as for usual consumption, often combining them into food composites or aggregates, homogenizing them, and analyzing them for toxic chemicals (WHO 1985; GEMS/Food 2005; EFSA/FAO/WHO 2011; Nasreddine et al. 2006). The preparation and analysis of food as consumed are essential since preparation techniques, including peeling, washing, and heating, are known to play a key role in reducing the levels of pesticide residues in foods (Schattenberg et al. 1996; Kaushik et al. 2009). This study’s findings will provide first estimates of dietary exposure to pesticide residues in Lebanon, shedding light on its magnitude and identifying its main contributors.

## Materials and methods

### Food consumption data

The types and quantities of foods that made up the average “urban total diet’ and the average “semi-rural total diet” are based on the results of previous studies, which are published elsewhere (Reshmany 2010; Nasreddine et al. 2010; Raad et al. 2014). Briefly, in each of the areas under investigation, food consumption data were collected by conducting individual food consumption surveys, following the same methodology, using the same dietary assessment instrument, and targeting the same population group, i.e., adults aged 25–54 years. The age and sex distribution of the study samples (*n* = 444 in Beirut and *n* = 200 in Keserwan) were proportionate to that of the baseline population, according to the National Survey of Household Living conditions (Central Administration for Statistics et al. 2006). In each area, the sample was drawn from randomly selected households. One adult from each household was invited to participate. The survey’s design and conduct were approved by the Institutional Review Board of the American University of Beirut, and informed consent from adults was obtained prior to enrolment in the study.

Food consumption data were collected by means of a culture-specific semi-quantitative food frequency questionnaire (FFQ) (Nasreddine et al. 2006). A reference portion, expressed in household measures or grams, was specified for each food item listed on the questionnaire.

The average individual’s daily ration, including drinking water and other drinks, was estimated at 3036.5 g/day for the urban area and of 3706.1 g/day for the semi-rural one.

### Food selection and collection of food samples

Two main criteria were applied when selecting the food items for analysis. First, given that the laboratory undertaking the analytical work has the capacity to analyze pesticide residues in low-fat food matrices, the selection of foods was limited to low-fat foods which included cereals, fruits, vegetables, as well as low-fat drinks including water, alcoholic beverages, fruit juices, and soft drinks. Applying this selection criterion resulted in the exclusion of animal-based products from the study. Coffee drinks were also excluded given that these beverages are usually consumed with high-fat creamers or high-fat milk. Second, food selection was based on a mean food consumption level exceeding 1 g/day/person, a criterion that resulted in the exclusion of canned hearts of palm from the urban diet (0.5 g/day), canned asparagus and canned mushrooms from the semi-rural diet (0.5 and 0.7 g/day, respectively), and breakfast cereals from both the urban and semi-rural diet (0.51 and 0.9 g/day, respectively).

Accordingly, 63 food items including drinking water were selected for the urban diet and 61 food items for the semi-rural one (Tables 1 and 2). On a weight basis, these foods represented 73.3 and 73.5 % of the average daily ration of the urban and semi-rural diets, respectively.

**Table 1:** Aggregation of the 63 food items into 21 food groups: weight of each item as consumed (g/day) and percentage weight of each food item in its group (urban diet)

Food group	Mean intake (g/day)	Percentage weight (%)
*1. Pulses:*		
Lentils	9.79	16
Chickpeas	12.7	21
Beans dry and cooked	5.31	9
Red beans	6.91	11
Fava beans	11.75	20
Green beans	13.72	23
Total	60.18	100
*2. Salads and raw vegetables:*		
Salad green	56.64	42
Tabbouli	12.93	10
Fattoush	15.55	11
Raw vegetables	49.36	37
Total	134.48	100
*3. Canned vegetables:*		
Mixed vegetables	3.22	32
Corn canned	4.61	45
Mushroom canned	1.19	12
Asparagus canned	1.09	11
Total	10.11	100
*4. Potatoes:*		
Boiled potato	63.51	82
Baked potato	13.73	18
Total	77.24	100
*5. Cooked green leafy vegetables:*		
Spinach and grape leaves	10.37	41.4
Chicory	3.34	13.3
Jew’s mellow	11.36	45.3
Total	25.07	100
*6. Brassica vegetables and artichoke:*		
Cooked and canned artichoke	3.77	25
Cooked cabbage	4.54	30
Cauliflower	6.69	45
Total	15	100
*7. Fruit bearing vegetables:*		
Cooked eggplant	8.73	34
Stuffed eggplant	3.9	15
Zucchini	10.95	43
Cooked okra	2.0	8
Total	25.58	100
*8. Melons:*		
Watermelons	15.04	78
Melons	4.2	22
Total	19.24	100
*9. Tropical fruits:*		
Exotic fruits	2.7	11
Dates	1.45	6
Bananas	20.69	83
Total	24.84	100
*10. Citrus:*		
Citrus	75.34	100
Total	75.34	100
*11. Pomes and stone fruits:*		
Apples	61	76
Apricot	2.1	3
Cherries	5.64	7
Peaches	5.32	6
Pears	5.24	6
Plums	1.39	2
Total	80.69	100
*12. Grapes and strawberries:*		
Grapes	10.04	74
Strawberries	3.47	26
Total	13.51	100
*13. Fruit salads:*		
Fruit salads	4.73	27
Fruit based desserts	10.95	61
Canned fruits	2.2	12
Total	17.88	100
*14. Breads:*		
Traditional bread	136.85	94
Kaak products	6.17	4
Toast and crackers	3.21	2
Total	146.23	100
*15. Pasta and bulgur:*		
Cooked pasta	23.96	74
Cooked bulgur	5.5	17
Uncooked bulgur	2.8	9
Total	32.26	100
*16. Rice and rice based products:*		
Cooked rice	50.1	100
Total	50.1	100
*17. Other cereal based products:*		
Manaeesh ^a^	32.13	40
Small pies ^a^	6.64	8
Pizza ^a^	11.28	14
Croissant	4.92	6
Cake ^b^	11.85	15
Biscuits	13.61	17
Total	80.43	100
*18. Fruit juices:*		
Fresh juice	65	50
Canned juice	65.16	50
Total	130.16	100
*19. Alcoholic beverages:*		
Beer	30.48	68
Wine	6.61	15
Others	7.8	17
Total	44.89	100
*20. Soft drinks:*		
Soft drinks	176.90	100
Total	176.90	100
*21. Water:*		
Water	985.95	100
Total	985.95	100

^a^For the traditional pies (manaeesh), small pies, and pizza, only the dough was bought without the filling which may be high in fat

^b^Cakes without cream were purchased

**Table 2:** Aggregation of the 61 food items into 21 food groups: weight of each item as consumed (g/day) and percentage weight of each food item in its group (semi-rural diet)

Food group	Mean intake (g/day)	Percentage weight (%)
*1. Pulses:*		
Lentils	14.76	19
Chickpeas	10.74	14
Beans dry and cooked	10.27	13
Red beans	9.81	12
Fava beans	9.19	12
Green beans	23.04	30
Total	77.81	100
*2. Salads and raw vegetables:*		
Salad green	64.58	33
Tabbouli	33.56	17
Fattoush	21.02	11
Raw vegetables	75.97	39
Total	195.13	100
*3. Canned vegetables:*		
Mixed vegetables	8.00	86
Canned corn	1.32	14
Total	9.32	100
*4. Potatoes:*		
Boiled potato	49.56	94
Baked potato	3.11	6
Total	52.67	100
*5. Cooked green leafy vegetables:*		
Spinach and grape leaves	14.54	42
Chicory	6.13	18
Jew’s mellow	13.77	40
Total	34.44	100
*6. Brassica vegetables and artichoke:*		
Cooked and canned artichoke	7.04	27
Cooked cabbage	11.54	45
Cauliflower	7.07	28
Total	25.65	100
*7. Fruit bearing vegetables:*		
Cooked eggplant	12.99	44
Stuffed eggplant	2.55	9
Zucchini	12	41
Cooked okra	1.84	6
Total	29.38	100
*8. Melons:*		
Watermelons	30.28	72
Melons	11.54	28
Total	41.82	100
*9. Tropical fruits:*		
Exotic fruits	6.7	18
Dates	1.81	5
Bananas	28.36	77
Total	36.87	100
*10. Citrus:*		
Citrus	53.42	100
Total	53.42	100
*11. Pomes and stone fruits:*		
Apples	72.66	52
Apricot	13	9
Cherries	22.05	16
Peaches	15.32	11
Pears	12.64	9
Plums	3.12	3
Total	138.79	100
*12. Grapes and strawberries:*		
Grapes	25.77	81
Strawberries	6.02	19
Total	31.79	100
*13. Fruit salads:*		
Fruit salads	7.81	33
Fruit based desserts	12.95	54
Canned fruits	3.14	13
Total	23.9	100
*14. Breads:*		
Traditional bread	127.41	95
Kaak products	4.86	4
Toast and crackers	1.82	1
Total	134.09	100
*15. Pasta and bulgur:*		
Cooked pasta	40.99	83
Cooked bulgur	5.5	11
Uncooked bulgur	2.8	6
Total	49.29	100
*16. Rice and rice based products:*		
Cooked rice	59.28	100
Total	59.28	100
*17. Other cereal based products:*		
Manaeesh ^a^	25.67	34
Small pies ^a^	5.63	7
Pizza ^a^	19.45	26
Croissant	1.39	2
Cake ^b^	11.86	16
Biscuits	12.23	15
Total	76.23	100
*18. Fruit juices:*		
Fresh juice	67.22	58
Canned juice	49.15	42
Total	116.37	100
*19. Alcoholic beverages:*		
Beer	43.91	66
Wine	12.37	19
Others	10.31	15
Total	66.59	100
*20. Soft drinks:*		
Soft drinks	99.37	100
Total	99.37	100
*21. Water:*		
Water	1371.72	100
Total	1371.72	100

^a^For the traditional pies (manaeesh), small pies, and pizza, only the dough was bought without the filling which may be high in fat

^b^Cakes without cream were purchased

The purchase and collection of the selected food items were conducted separately for each of the two areas under investigation (i.e., urban vs semi-rural total diet). Based on the general guidelines provided by the WHO (WHO 1985) and on the sampling schemes described in the literature (Leblanc et al. 2005), the composite sampling approach was applied in this study and consisted of purchasing the same item from five different sites or in five different brands/varieties and combining the five items to represent a composite sample of the food product in question. Since market shares of the different brands/varieties are not available in Lebanon, the contribution of each sub-sample to total weight was equal to 20 %. Even though the composite sampling scheme may dilute high contamination levels that can be found in one of the collected sub-samples, it has the advantage of increasing the representativeness of food sampling (WHO 1985). The purchase and collection of foods took place in 2010. In order to take into account the occurrence of changeable contamination levels, three complete sets of foods (market baskets) were collected from each area at 3-month intervals, and the sampling was performed at the most popular retail outlets in each of the studied areas.

Subsequently for the urban area, 63 food items × five subsamples × three MB = 945 food samples were collected, while for the rural area, 61 food items × five sub-samples × three MB = 915 food samples were collected. Accordingly, a total of 1860 food samples were collected for this study. For each food item, 1 kg of each subsample (i.e., variety) was collected.

### Preparation of the foods “as consumed” and aggregation of samples

The collected food samples were transported to one central laboratory (Pilot Plant/Department of Nutrition and Food Sciences, American University of Beirut) within hours after collection, where they were prepared as for normal consumption (i.e., washing, peeling, and simple cooking procedures when applicable). The water used for cooking purposes was specific to each of the areas under study, i.e., Beirut or Kesrwan. During the preparation of the items to the table ready form, suitable stainless steel equipment was used thus excluding plastic containers and utensils. For the traditional pies (manaeesh), small pies, and pizza, the dough was purchased without the filling which is usually high in fat.

For each of the three market baskets and for each of the selected food items (i.e., 63 for the urban diet and 61 for the rural diet), the five sub-samples were combined (20 % *w*/w) and blended to give a homogeneous sample that represents the food item in question. To minimize degradation of certain pesticide residues, the samples were homogenized at low temperature (i.e., in the frozen state) (Pihlström et al. 2008), in a stainless steel blender. In accordance with good laboratory practices and in compliance with the ISO/IEC 17025 norm (UNIDO 2009), the equipment used for the preparation and homogenization of the composite samples was thoroughly washed between each preparation (e.g., cleaning with a laboratory-grade detergent, rinsing thoroughly with hot tap water, rinsing or soaking with acid solution, rinsing thoroughly with deionized water) to avoid the risk of cross-contamination.

For each of the three market baskets and for each of the urban and semi-rural “total diet,” food items were aggregated into 21 groups of similar foods for the analysis of pesticide residues. The aggregation of fruits and vegetables was based on the WHO/Food and Agriculture Organization (FAO) classification of crops (FAO 2005). The appropriate amount of each raw, prepared, or cooked food item to be included in its composite food group was determined based on the respective food consumption data (Tables 1 and 2). Food items of each group were combined and homogenized, while frozen, using a stainless steel blender.

Therefore, for the urban diet, 21 composite food groups per market basket x three market baskets = 63 composite food group samples were prepared for analysis. Similarly, and following the same procedure for the semi-rural diet, 63 composite food group samples were prepared, thus amounting to 126 samples prepared for analysis in this study. Composite samples were then stored at −20 °C overnight in sealed disposable aluminum foil containers for solid foods or in glass bottles for liquids, until their delivery in ice to the laboratory for chemical analysis, the next morning,

### Analytical determination of pesticide residues in food samples

The analysis of pesticide residues was conducted in the Laboratory of Analysis of Organic Compounds (Lebanese Atomic Energy Commission, National Council for Scientific research), assigned by the Lebanese Ministry of Agriculture and the Lebanese Ministry of Economy for the analysis of organic pollutants, such as pesticide residues, in food items and water. In 2010, this laboratory had the analytical capacity to analyze 47 different compounds (parent pesticides and their metabolites) in low-fat food matrices. The following 47 pesticides were included in the study:

**Table:** 

Alachlor	o,p’-DDE	Gamma HCH	Pirimicarb
Aldrin	Dieldrin	Delta HCH	Primiphos methyl
Bifenthrin	Dimethoate	Hexachlorobenzene	Procymidone
Buprofezin	Endosulfan sulfate	Heptachloro-exo-epoxide	Propoxur
Carbaryl	Alpha endosulfan	Heptachloro-endo-epoxide	Tecnazene
Chlorpyrifos	Beta endosulfan	Kresoxim-methyl	Tetradifon
Chlorpyrifos methyl	Ethion	Malathion	Tolclofos-methyl
Diazinon	Ethoprophos	Methacrifos	Trifluralin
p,p’-DDT	Etrimfos	Methoxychlor	
o,p’-DDT	Fenitrothion	Quintozene	
p,p’-DDD	Fludioxonil	Parathion	
o,p’DDD	Alpha Hexachlorocyclohexane (HCH)	Parathion-Methyl	
p,p’-DDE	Beta HCH	Pendimethalin	

The analysis of the pesticide residues was performed on all the collected food samples, since, in Lebanon, the use of pesticides is not clearly regulated by types of crops.

### Chemicals

Pesticide standards of high purity level (98–99 %) were purchased from Dr. Ehrenstorfer GmbH (Augsburg, Germany) and ChemService (West Chester, PA, USA). Individual standard pesticide solutions were prepared in acetonitrile with a concentration of about 1000 mg/L. Standard mixture pesticide solutions were prepared by diluting each individual standard solution with acetonitrile, in order to get a concentration of about 35 mg/L for each compound. Working standard solutions were prepared by diluting the standard mixture pesticide solutions with acetonitrile. All standard solutions were stored at a temperature below −20 °C.

### Extraction method

The analytical determination of pesticide residues was carried out using the QuEChERS Multiresidue Method (Anastassiades et al. 2003) in the Laboratory of Organic Pollutants, Lebanese Atomic Energy Agency, National Council for Scientific Research)**.** Ten grams of the frozen food sample was weighed into a 50-mL centrifuge tube to which 10 mL and 100 μL of acetonitrile and PCB52 (internal standard) were added, respectively. The mixture was then shaken vigorously for 1 min to ensure the extraction of pesticides from the matrix and a mixture of 4 g MgSO_4_,_,_ 1 g NaCl, 1 g Na_3_ citrate dehydrate, and 0.5 g Na_2_H citrate sesquihydrate was added. After the addition of each salt, the tube was shaken strongly for 1 min before centrifuging the mixture of salts for 5 min at 3000 U/min. Samples with low water content such as breads and other cereal-based products were prepared by initially weighing 5 g of sample instead of 10 g and adding 10 mL of water.

For the purification step, 5 mL of the extracts was transferred into a PP single use centrifugation tube, which contains (5*25 = 125 mg) PSA and (5*150 = 750 mg) MgSO_4_ and then shaken well for 30 s before being centrifuged for 5 min at 3000 U/min.

After purification, 1 mL of each extract was transferred into a screw cup vial and acidified with 10 μL of 5 % formic acid in acetonitrile. The cleaned and acidified extracts were then transferred into auto-sampler vials and used for the multi-residue determination by GC/MS.

### Gas chromatography coupled to mass spectrometer analysis

An Agilent 6890N GC connected to an Agilent 5975 MS was used (Agilent technologies, USA). The GC-MS was equipped with an Agilent 7683 B auto sampler and split/ splitless injector with electronic pressure control. The used column was a capillary column (HP-5MS, 30 m, 0.25 mm i.d., 0.25 μm, Agilent J & W GC columns). The temperature program was the following: initial temperature 70 °C, held for 1 min, 10 °C/min ramp to 160 °C then held for 5 min, finally by 3 °C/min to 240 °C and held for 18.5 min. The total analysis time was 60.17 min and the equilibration time 0.5 min. The temperature of the injection port was 250 °C, and a 1-μL volume was injected in splitless mode. Helium was used as carrier gas at a constant flow of 1 mL/min. The mass spectrometer was operated in electron ionization mode with an ionizing energy of 70 eV, ion source temperature 230 °C, MS quadruple temperature 150 °C, and solvent delay 3 min.

Analysis was performed in the selected ion monitoring (SIM) mode based on the use of one target and two or three qualifier ions. Target and qualifier abundances were determined by the injection of individual pesticide standards under the same chromatographic conditions in full-scan mode with the mass/charge ratio ranging from m/z 50 to 550. Pesticides were identified according to the retention times, the target and qualifier ions, and the qualifier to target abundance ratios (Kouzayha et al. 2013).

### Analytical quality control

All data were subjected to strict quality control procedures, such as the analysis of procedural and instrumental blanks in addition to spiked samples with each set of analyzed samples. The target pesticide quantification was based on peak area ratio of the target ion divided by the internal standard (PCB 153). Surrogate standard (PCB 52) recoveries were calculated to monitor the analytical process’s performance, and the obtained values were higher than 60 %.

For all pesticide residues under investigation, the limit of detection (LOD) was of 3.33 μg/kg of sample matrix except for beta endosulfan, parathion, and heptachloro-endo-epoxide, for which the LOD was of 13.33 μg/kg. For all pesticide residues under investigation, the limit of quantification (LOQ) was of 10 μg/kg of sample matrix except for beta endosulfan, parathion, and heptachloro-endo-epoxide, for which the LOQ was of 40 μg/kg.

### Calculation of dietary exposure

Calculation of dietary exposure was performed for those pesticide residues that were detected or quantified in at least one food sample in at least one of the areas under study (Beirut or Kesrwan).

The deterministic approach was applied for the calculation of the dietary exposure to pesticide residues. Given that the percentage of censored data (results reported below LOD and/or LOQ) exceeded 60 % for all the pesticide residues, two scenarios were adopted: (1) the lower bound (LB) approach by replacing the results below LOD by zero and results below LOQ by LOD and (2) the upper bound (UB) approach by replacing the results below LOD by LOD and results below LOQ by LOQ (GEMS/Food-Euro 1995; EFSA 2010).

To take into account the variability that exists in food consumption patterns and to provide data on the exposure levels’ distribution within the studied population, the distributions of food intakes as provided by the individual dietary surveys were combined with the average concentration for each pesticide residue under study. Accordingly, mean and 95 percentile exposure levels (p95) were computed, and risk characterization was performed for average and high exposure levels. To compare dietary exposure levels with the respective ADIs which are expressed per kg body weight, an average body weight of 72.8 kg was adopted for the urban population since it was the average weight of the participants in the food consumption survey (Nasreddine et al. 2010) and an average body weight of 73.6 kg for the rural population (Reshmany 2010).

Dietary exposure levels at both the LB and UB scenarios were calculated using the Statistical Analysis Package for Social Sciences, version 18.0 (SPSS Inc., Chicago, IL, USA). Dietary exposure levels were first expressed in micrograms of residue per kilogram of body weight per day (μg/kg bw/d), then as a percentage of the ADI in order to characterize the chronic risk. The ADI may apply, in certain instances, to the sum of specific chemicals, and not to a single chemical. For instance, the ADI is applicable to DDT, not to the individual residues o,p’-DDD, p,p’-DDD, o,p’-DDE, p,p’-DDE, o,p’-DDT, p,p’-DDT.

The contributions of each food group to the dietary exposure to each pesticide residue were also calculated.

## Results and discussion

### Substances detected and their levels in the analyzed composite food samples

In this study, the dietary exposure of Lebanese adults to 47 pesticide residues was evaluated based on the consumption of drinks and foods of plant origin. The list of analyzed pesticide residues comprises 22 priority pesticide residues as per the priority list of the GEMS/Food environmental surveillance program (WHO 2006). The analyzed residues include pesticides that are currently authorized and are the most commonly used in the country in addition to older pesticides, the use of which has been banned as of 1998 (Ministry of Environment (Lebanon) 1998) (i.e., persistent organic pollutants, POPs). However, the banned pesticides may still be detected in certain food items available on the local market given that they tend to linger in the environment and that these banned chemicals may still be illegally entering Lebanon due the absence of effective law enforcement mechanisms (Ministry of Environment (Lebanon) et al. 2005).

Table 3 shows the frequency of detection and quantification as well as the minimum, maximum, and mean estimated levels (LB and UB) for each *food group-substance* combination positive to screening. Out of the 47 substances studied, 18 residues (38.2 %) were detected/quantified on at least one composite sample and included *Procymidone, alpha endosulfan, beta endosulfan, endosulfan sulfate, fenithrion, malathion, dieldrin, chlorpyrifos, ethion, Dimethoate, chlorpyrifos methyl, gamma HCH, primiphos methyl, Propoxur, DDDppp, fluodioxonil, kresoxim methyl, and diazinon*. All of the detected/quantified residues are pertaining to pesticides that are mainly used as insecticides, except for *procymidone, fluodioxonil, and kresoxim methyl,* which are used as fungicides, and *malathion,* which is used as an insecticide and acaricide.

**Table 3:** Analytical results summarized by food group-pesticide combination with at least one detection in each of the areas under investigation (Kesrwan/semi-rural diet and Beirut/urban diet) ^a^

Food group	Pesticide residue	Frequency of detection	Frequency of quantification	Minimum quantified value (μg/kg)	Maximum quantified value (μg/kg)	Mean estimated level (LB) (μg/kg)	Mean estimated level (UB) (μg/kg)
Semi-rural diet							
Salads and raw vegetables	Procymidone	33.3 %	0 %	----	----	1.11	5.55
	Edosulfan Sulfate	33.3 %	33.3 %	12.33	12.33	5.22	8.55
Brassica vegetables and artichoke	Fenitrothion	33.3 %	0 %	----	----	1.11	5.55
	Malathion	33.3 %	0 %	----	----	1.11	5.55
Canned vegetables	Dieldrin	33.3 %	0 %	----	---	1.11	5.55
Fruit bearing vegetables	Procymidone	33.3 %	33.3 %	----	----	1.11	5.55
	Chlorpyrifos	0 %	33.3 %	48.93	48.93	16.31	18.53
Fruit juices	Chlorpyrifos	33.3 %	0 %	-----	-----	1.11	5.55
	Ethion	33.3 %	0 %	-----	----	1.11	5.55
Fruit salads	Chlorpyrifos	0 %	100 %	11.5	24.51	16.6	16.6
	Dimethoate	33.3 %	0 %	----	----	1.11	5.55
Grapes and strawberries	Chlorpyrifos	0 %	33.3 %	18.83	18.83	6.27	8.49
	Chlorpyrifos methyl	0 %	33.3 %	10.8	10.8	3.6	5.82
Tropical fruits	Dieldrin	0 %	33.3 %	30	30	10	12.22
	Diazinon	33.3 %	0 %	----	----	1.11	5.55
Pome and stone fruits	Chlorpyrifos	66.6 %	33.3 %	82.31	82.31	29.6	34.1
	Dimethoate	33.3 %	0 %	----	---	1.11	5.55
Cooked green leafy vegetables	Dieldrin	33.3 %	0 %	----	-----	1.11	5.55
	Chlorpyrifos	33.3 %	66.6 %	11	208	74.1	76.3
	Kresoxim-methyl	0 %	33.3 %	159.9	159.9	53.3	55.55
	Propoxur	0 %	33.3 %	10.7	10.7	3.6	5.7
Pasta and bulgur	Gamma HCH (lindane)	33.3 %	0 %	----	----	1.11	5.5
	Primiphos methyl	33.3 %	33.3 %	22	22	8.4	11.77
	DDD,pp	0 %	33.3 %	15	15	5	7.22
Other cereal-based products	Chlorpyrifos	0 %	33.3 %	20	20	6.66	8.88
	Primiphos methyl	0 %	33.3 %	21	21	7	9.22
Urban diet							
Salads and raw vegetables	Procymidone	0 %	33.3 %	45.28	45.28	15.1	17.33
Brassica vegetables and artichoke	Procymidone	0 %	33.3 %	13.5	13.5	4.5	6.72
	Dimethoate	0 %	33.3 %	25.85	25.85	8.6	10.8
Fruit bearing vegetables	Procymidone	33.3 %	33.3 %	18.95	18.95	7.4	9.6
Fruit salads	Propoxur	0 %	33.3 %	10.5	10.5	3.5	5.7
	Fludioxonil	0 %	33.3 %	22.52	22.52	7.5	9.72
Grapes and strawberries	Procymidone	0 %	33.3 %	13.96	13.96	4.65	6.87
	Alpha-endosulfan	0 %	33.3 %	52.73	52.73	17.6	19.8
	Beta-endosulfan	0 %	33.3 %	75.77	75.77	25.2	34.14
	Chlorpyrifos	0 %	33.3 %	33.62	33.62	11.2	13.4
Pome and stone fruits	Chlorpyrifos	0 %	33.3 %	57.26	57.26	19.08	21.3
	Dimethoate	0 %	33.3 %	74.56	74.56	24.8	27.07
Cooked green leafy vegetables	Chlorpyrifos	0 %	66.6 %	34.55	158.6	64.38	65.49
	Kresoxim-methyl	0 %	33.3 %	55.87	55.87	18.62	20.84
	Propoxur	0 %	33.3 %	10.7	10.7	3.6	5.8
Pasta and bulgur	Primiphos methyl	0 %	33.3 %	20.22	20.22	6.74	8.96
Rice and rice-based products	Primiphos methyl	0 %	33.3 %	24.69	24.69	8.23	10.45
Breads	Primiphos methyl	33.3 %	0 %	----	----	1.11	5.55
	Dimethoate	0 %	33.3 %	20.64	20.64	6.88	9.1
	Chlorpyrifos methyl	0 %	33.3 %	10.3	10.3	3.43	5.65

For all pesticide residues listed in the table above, LOD = 3.33 μg/kg except for beta endosulfan for which LOD = 13.33 μg/kg

For all pesticide residues listed in the table above, LOQ = 10 μg/kg except for beta endosulfan for which LOQ = 40 μg/kg

^a^Three market baskets were collected, and as such, three samples were analyzed per food group per area

Although the analytical limits may not have permitted the detection of trace levels of some residues, the LODs in the present study are lower than the ones used by recent TDSs, such as the one conducted in Cameroon (5 μg/kg) (Gimou et al. 2008), and within the range of those adopted by the French TDS (1–7 μg/kg for the group of pesticide residues that are in common with the present study) (Nougadère et al. 2012). The fact that the other investigated residues were not detected in food samples may be explained by the possible dilution of potential residual levels due to the composite nature of the samples (Nougadère et al. 2012). It is important to note that the preparation techniques of foods as consumed (peeling, washing, and cooking), which are suggested to decrease contamination levels, may also explain the low or undetectable levels of some pesticide residues in the present study (Kaushik et al. 2009; Rasmusssen et al. 2003).

Out of the 126 composite samples analyzed, 42 (33.3 %) registered at least one detectable residue. No residues were detected in the following food groups: pulses, potatoes, melons, citrus, alcoholic beverages, soft drinks, and water in both the urban and semi-rural diet. Out of the 42 composite samples in which one or more residues were detected, 55 % had only one residue, and 45 % had 2 to 4 residues. No more than 4 residues were detected in a single sample. Regarding the semi-rural diet, the highest number of residues was detected in “cooked green leafy vegetables” (4 residues) and fruit juices (4 residues) followed by “pasta and bulgur” (3 residues). As for the urban diet, the food group “grapes and strawberries” registered the highest number of residues (4), followed by “cooked green leafy vegetables” (3) and “breads” (3). The concomitant presence of several pesticide residues in some composite food samples has been also reported by Nougadère et al. (2012) in the French TDS. Additional investigations are needed with regards to the exposure to pesticide mixtures and their combined health effects given the potential synergy that some of these residues may have in impacting human health (Reffstrup et al. 2010; EFSA 2009).

Of all the analytical results (i.e., all substance-sample combinations), 5856 (99 %) were associated with undetected residues (<LOD), while 57 (1 %) were associated with detectable/quantifiable residues. These results are similar to those reported by Nougadère et al. (2012) based on the French TDS, where 99.3 % of analytical results were associated with undetected residues (<LOD). Out of the 57 results associated with detected residues, 19 (33.3 %) were detectable and 38 (66.7 %) quantifiable. The most frequently detected/quantified pesticide residues in the semi-rural diet included Chlorpyrifos (37.1 % of composite samples containing detectable or quantifiable residue levels), followed by Dieldrin (8.6 %), Procymidone (8.6 %), and Primiphos methyl (8.6 %). As for the urban diet, the most frequently detected/quantified residues also included Procymidone (22.7 %) followed by Chlorpyrifos (18.2 %), Primiphos methyl (13.6 %), and Dimethoate (13.6 %). When looking at quantifiable results only, the most frequently quantified pesticide residues in the semi-rural diet were Chlorpyrifos (50 % of quantifiable results) and Primiphos methyl (11.1 %), whereas in the urban diet, Chlorpyrifos (20 %), Procymidone (20 %), and Dimethoate (15 %) were the most frequently quantified residues.

In the urban diet, the quantified residues’ levels ranged between 10.3 and 158.6 μg/kg of food, whereas the quantified residues’ levels in the semi-rural diet ranged between 10.7 and 208 μg/kg of food. All the detected substances were authorized for agricultural purposes in Lebanon during the sampling period (2010), with the exception of the five POPs (Ministry of Environment (Lebanon) 1998). Lindane was detected in a composite sample of “pasta & bulgur,” and DDD,pp. was quantified in another composite sample of “pasta & bulgur” (15 μg/kg) from the semi-rural diet. Similarly, Dieldrin was detected in two composite samples (“canned vegetables” and “cooked green leafy vegetables”) and quantified in one composite sample of “tropical fruits” (30 μg/kg) from the semi-rural diet. It is noteworthy that the use of Dieldrin, DDT, and Lindane has been banned in Lebanon since 1998 (Ministry of Environment (Lebanon) 1998). The detection of these residues in some of the analyzed food samples may be due to POPs’ historical uses and persistence in the environment (Nougadère et al. 2012). Alternatively, the non-enforcement of regulatory measures in the country may implicate an illicit use of these pesticides in some regions in Lebanon (Abu Jawdeh and Lebanese Environmental Forum (LEF) 2006). For instance, a study conducted on sediment samples in the North of Lebanon has documented (IDRC 2003) measurable amounts of DDT and DDE in all of the collected samples, suggesting current use of DDT parent compounds, albeit a banned substance. In addition, the active ingredients of technical endosulfan (α-endosulfan and β-endosulfan) were quantified in one composite sample of “grapes and strawberries” from the urban diet, while, in the semi-rural diet, endosulfan sulfate was detected in one composite sample of “salads and raw vegetables” and quantified in another (12.33 μg/kg: salads and raw vegetables). In 2011, endosulfan was listed in Annex A of the Stockholm Convention on Persistent Organic Pollutants (Stockholm Convention 2011), and its use was banned in Lebanon as of February 2010 (decree 1/79, dated 16/02/2010, Ministry of Agriculture, Lebanon), a period coinciding with the food sampling undertaken in the present study.

Although MRLs do not directly apply to composite samples and mixed dishes, the residue levels obtained in this study have been compared to the MRLs of the corresponding raw commodities, derived from the European Union Pesticide Database (European Commission 2010). Exceedances of the MRLs were observed in seven composite samples (16.6 % of the samples with detectable residues): 4 from the urban diet and 3 from the semi-rural one. These exceedances were for instance observed for Dimethoate in “Pome and stone fruits” (urban diet), Chlorpyrifos and Kresoxim-methyl in “Cooked green leafy vegetables” (both urban and rural diets) (European Commission 2010). In the composite sample of tropical fruits which includes bananas and exotic fruits such as mango and pineapple, the levels of Dieldrin were also found to exceed the MRL for “large fruits with inedible peels” (30 vs 10 μg/kg). Similarly, the level of alpha endosulfan and beta endosulfan in the composite sample of “grapes and strawberries” (alpha endosulfan: 52.73 μg/kg and beta endosulfan: 75.77 μg/kg; urban diet) were found to exceed the respective MRL (50 μg/kg for grapes and strawberries). Since the exact origin of the products (country of production) was unknown, and since the same sample is likely to include different products from different sources, it would not be possible to explain the origin of these exceedances (Nougadère et al. 2012). In order to better understand these non-conformities, it is necessary to set up, in Lebanon, robust surveillance and monitoring systems relevant to pesticide residues’ levels. Nevertheless, it should be kept in mind that the MRL is not a toxicological limit and therefore its exceedance cannot be directly extrapolated to human health risks.

In this study, pesticide residues were not detected in any of the analyzed beverages. In agreement with our results, Nougadère et al. (2012) have shown that none of the pesticide residues investigated in the present study (*n* = 47) were detected in soft drinks and drinking water samples in the French TDS, whereas only fludioxonil was detected in alcoholic beverages.

### Dietary exposure and risk characterization

In this study, dietary exposure assessment was performed using two scenarios: (1) the lower bound approach (LB) and (2) the upper bound approach (UB). Since the LB approach systematically assigns zero to non-detects, it tends to underestimate exposure, reflecting an optimistic scenario (Kettler et al. 2015). On the other hand, since the UB approach systematically assigns LOD/LOQ to non-detects, it tends to overestimate exposure, reflecting the worst case scenario (Kettler et al. 2015). As such, the calculation of lower and upper bound exposure estimates aims at illustrating the variation between an optimistic and a worst case scenario (Kettler et al. 2015).

Using the LB approach, the highest mean estimated daily exposures (>1 μg/day) were observed for Chlorpyrifos (3.35 μg/day) followed by Dimethoate (3.14 μg/day) and Procymidone (2.32 μg/day) in the urban diet, whereas the highest mean exposure estimates were observed for Chlorpyrifos (9.28 μg/day), Kresoxim methyl (1.85 μg/day), and endosulfan (1.25 μg/day) in the semi-rural diet. For all the residues under investigation, mean estimated daily exposures were far below the ADIs. The pesticide residues with the highest contribution to ADIs (>1 % of ADI) were Dieldrin (5.5 %) and Chlorpyrifos (1.26 %) in the semi-rural diet and Dimethoate in the urban diet (4.3 %). The 95th exposure levels for these pesticide residues reached 7.8 % of the ADI for Dimethoate (urban diet), 15.9 % for Dieldrin, and 2.89 % for chlorpyrifos (semi-rural diet). Under the LB approach, the proportion of subjects exceeding the ADIs was nil for the 18 residues in both the urban and semi-rural diets. It is important to note that, to allow comparison with previous TDSs, the ADI for chlorpyrifos used in Tables 4 and 5 is based on the 2005 toxicological evaluation of this substance (JMPR 2008), which was set at 0.01 mg/kg bw/day. However in 2014, based on new toxicological data documenting a significant decrease in Red Blood Cell Cholinesterase in rats, the ADI for Chlorpyrifos was reduced to 0.001 mg/kg bw/day (EFSA 2014). When adopting this ADI, mean estimated daily exposure to chlorpyrifos (LB) was found to contribute 4.6 % of the ADI in the urban diet and 12.61 % in the semi-rural diet, whereas the 95th exposure levels were found to contribute 8.36 % of the ADI in the urban diet and 28.9 % in the semi-rural diet.

**Table 4:** Estimated mean and 95th dietary exposure levels to pesticide residues (μg/day) that were detected/quantified in at least one food sample and contribution of dietary exposure to acceptable daily intakes (ADIs): lower bound estimates

Pesticide residue	Urban population					Semi-rural population				
	Mean		95th percentile		Subjects exceeding ADI	Mean		95th percentile		subjects exceeding ADI
	Mean ± SD (μg/d)	%ADI	μg/d	%ADI	%	Mean ± SD (μg/d)	% ADI	μg/d	% ADI	%
Procymidone	2.32 ± 0.97	0.1138	4.13	0.20	0	0.25 ± 0.17	0.0121	0.61	0.03	0
Endosulfan^c^	0.58	0.1328	1.06	0.24	0	1.25	0.2831	3.14	0.71	0
Fenitrothion	0	0	0	0	0	0.03 ± 0.04	0.0082	0.10	0.03	0
Malathion	0	0	0	0	0	0.03 ± 0.04	0.0014	0.10	0.0045	0
Dieldrin	0	0	0.00	0	0	0.41 ± 0.39	5.5707	1.17	15.90	15.8
Chlorpyrifos	3.35 ± 1.60	0.4602	6.09	0.84	0	9.28 ± 6.80	1.2609	21.27	2.89	0
Ethion	0	0	0.00	0	0	0.13 ± 0.40	0.0883	0.54	0.37	0
Dimethoate	3.14 ± 1.37	4.3132	5.71	7.84	0	0.18 ± 0.21	0.2446	0.44	0.60	0
Chlorpyrifos-methyl	0.49 ± 0.22	0.0673	0.83	0.11	0	0.03 ± 0.04	0.0041	0.11	0.0149	0
Gamma HCH	0	0	0.00	0	0	0.06 ± 0.06	0.0163	0.16	0.04	0.0435
Primiphos methyl	0.79 ± 0.38	0.2713	1.44	0.49	0	0.97 ± 0.71	0.3295	2.30	0.78	0.7813
Propoxur	0.029 ± 0.18	0.0020	0.58	0.04	0	0.12 ± 0.14	0.0082	0.39	0.03	0.0265
p,p’-DDD^d^	0	0	0	0	0	0.26 ± 0.29	0.0353	0.70	0.0951	0.0951
Fludioxonil	0.13 ± 0.16	0.0005	0.41	0.0015	0	0.00	0	0	0	0
Kresoxim-methyl	0.48 ± 0.30	0.0016	0.97	0.0033	0	1.85 ± 2.02	0.0063	5.80	0.0197	0.0197
Diazinon	0	0	0.00	0	0	0.07 ± 0.07	0.4755	0.23	1.5625	1.5625

An average body weight of 72.8 kg was used for the urban population and of 73.6 kg for the semi-rural population

**ADI**s: Chlorpyrifos: 0.01 mg/kg bw/day (JMPR 2008); Chlorpyrifos-methyl: 0.01 mg/kg bw/day (JMPR 2013); Diazinon: 0.0002 mg/kg bw/day (EFSA 2006a); Dieldrin: 0.0001 mg/kg bw/day (JMPR 2008, 1995); Dimethoate: 0.0010 mg/kg bw/day (EFSA 2006b); Ethion: 0.002 mg/kg bw/day (JMPR 2008); Fenitrothion: 0.005 mg/kg bw/day (JMPR 2008; EFSA 2006c); Fludioxonil: 0.37 mg/kg bw/day (EFSA 2007); Gamma HCH: 0.005 mg/kg bw/day (JMPR 2008); Kresoxim-methyl: 0.4 mg/kg bw/day (JMPR 2008); Malathion: 0.03 mg/kg bw/day (EFSA 2009); Pirimiphos-methyl: 0.004 mg/kg bw/day (EFSA 2005); Procymidone: 0.0280 mg/kg bw/day (ESFA 2011); Propoxur: 0.02 mg/kg bw/day (JMPR 2008)

^a^Exposure estimate to endosulfan is based on the sum of alpha- and beta-endosulfan in the urban diet while it is based on endosulfan sulfate in the semi-rural diet. ADI for endosulfan: 0.006 mg/kg bw/day (JMPR 2008);

^b^The ADI is for DDT: 0.01 mg/kg bw/day (JMPR 2001, 2008) rather for the specific sub-residues such as p,p’-DDD

**Table 5:** Estimated mean and 95th dietary exposure levels to pesticide residues (μg/day) that were detected/quantified in at least one food sample and contribution of dietary exposure to acceptable daily intakes (ADIs): upper bound estimates

Pesticide residue	Urban population					Semi-rural population				
	Mean		95th percentile		subjects exceeding ADI	Mean		95th percentile		subjects exceeding ADI
	Mean ± SD (μg/day)	%ADI	μg/day	%ADI	%	Mean ± SD (μg/day)	% ADI	μg/day	% ADI	%
Procymidone	9.43 ± 2.09	0.46	13.11	0.64	0	9.55 ± 4.43	0.46	18.66	0.91	0
Endosulfan^c^	28.24 ± 5.14	6.47	39.82	9.12	0	55.35 ± 21.61	12.53	107.72	24.39	0
Fenitrothion	7.33 ± 1.71	2.01	10.28	2.82	0	9.10 ± 4.31	2.47	17.82	4.84	0
Malathion	7.33 ± 1.71	0.34	10.28	0.47	0	9.10 ± 4.31	0.41	17.82	0.81	0
Dieldrin	7.33 ± 1.71	100.69	10.28	141.21	46.4	9.47 ± 4.42	128.67	18.21	247.42	64.7
Chlorpyrifos	10.38 ± 2.42	1.43	14.83	2.04	0	17.40 ± 9.53	2.36	33.46	4.55	0
Ethion	7.33 ± 1.71	5.03	10.28	7.06	0	9.30 ± 4.63	6.32	18.11	12.30	0
Dimethoate	10.49 ± 2.38	14.41	14.70	20.19	0	9.41 ± 4.44	12.79	18.53	25.18	0
Chlorpyrifos- methyl	7.65 ± 1.78	1.05	10.55	1.45	0	9.12 ± 4.32	1.24	17.84	2.42	0
Gamma HCH	7.33 ± 1.71	2.01	10.28	2.82	0	9.16 ± 4.31	2.49	17.77	4.83	0
Primiphos methyl	8.19 ± 1.89	2.81	11.66	4.00	0	9.49 ± 4.39	3.22	18.03	6.12	0
Propoxur	7.43 ± 1.73	0.51	10.44	0.72	0	9.13 ± 4.32	0.62	17.91	1.22	0
p,p’-DDD^d^	7.33 ± 1.71	1.01	10.28	1.41	0	9.25 ± 4.33	1.26	17.84	2.42	0
Fludioxonil	7.44 ± 1.74	0.03	10.54	0.04	0	9.05 ± 4.29	0.03	17.76	0.07	0
Kresoxim-methyl	7.78 ± 1.77	0.03	10.76	0.04	0	10.86 ± 5.29	0.04	20.42	0.07	0
Diazinon	7.33 ± 1.71	50.34	10.28	70.6	0.5	9.12 ± 4.32	61.96	17.84	121.20	10

An average body weight of 72.8 kg was used for the urban population and of 73.6 kg for the semi-rural population

**ADIs**: Procymidone: 0.0280 mg/kg bw/day (ESFA 2011); Dieldrin: 0.0001 mg/kg bw/day; Chlorpyrifos: 0.0100 mg/kg bw/day (JMPR 2008); Chlorpyrifos-methyl: 0.0100 mg/kg bw/day (JMPR 2013); Diazinon: 0.0002 mg/kg bw/day (EFSA 2006a); Dimethoate: 0.0010 mg/kg bw/day (EFSA 2006b); Ethion: 0.0020 mg/kg bw/day (JMPR 2008); Fenitrothion: 0.0050 mg/kg bw/day (JMPR 2008; EFSA 2006c); Fludioxonyl: 0.3700 mg/kg bw/day (EFSA 2007); Kresoxim-methyl: 0.4000 mg/kg bw/day (JMPR 2008); Pirimiphos-methyl: 0.0040 mg/kg bw/day (EFSA 2005); Propoxur: 0.0200 mg/kg bw/day (JMPR 2008); Malathion: 0.0300 mg/kg bw/day (EFSA 2009); Gamma HCH: 0.005 mg/kg bw/day (JMPR 2008);

^a^Exposure estimate to endosulfan based on the sum of alpha- and beta-endosulfan in the urban diet while it is based on endosulfan sulfate in the semi-rural diet. ADI for endosulfan: 0.006 mg/kg bw/day (JMPR 2008);

^b^The ADI is for DDT: 0.01 mg/kg bw/day (JMPR 2001, 2008) rather for the specific sub-residues such as p,p’-DDD

Using the upper bound approach, which tends to overestimate dietary exposure, mean estimated daily exposures were found to range between 7.33 and 55.35 μg/day, with the highest value being observed for endosulfan. The estimated daily exposure exceeded 1 % of the respective ADIs, for all the residues, except for Procymidone, Malathion, Propoxur, Fludioxonil, and Kresoxim-methyl. The mean exposure estimate for Dieldrin was found to exceed the ADI in the semi-rural diet, thus representing 128.7 % of the ADI and to be equivalent to the ADI in the urban diet (100.7 %). Mean estimated daily exposure for Diazinon was found to reach 50.3 % of the ADI in the urban diet and 61.9 % in the semi-rural diet. Using the updated ADI for Chlorpyrifos, mean estimated daily exposure was found to contribute 14.3 and 24 % in the urban and semi-rural diets, respectively. It is important to note that, since pesticide residues were not detected in any sample of beverages in the present study, the use of the UB approach may particularly overestimate exposure levels, given the high intake levels of beverages per day and the attribution of LOD and LOQ values to the undetected or unquantified results. As such, the UB dietary exposure was recalculated, without considering the beverages (data not shown). With this scenario, the mean estimated daily exposure to Dieldrin was found to be close to half the ADI in both Beirut and the semi-rural area of Kesrwan and to only exceed the ADI under the 95th percentile exposure level in the semi-rural setting (124 % of the ADI). As for Diazinon, both the average and 95th exposure levels did not exceed 30 % of the ADI except for the semi-rural area where the 95th exposure level reached 56 % of the ADI (data not shown).

Even though the present study does not include a complete coverage of the total diet, its results are compared to those provided by TDSs conducted during the same period of time (Gimou et al. 2008; Nougadère et al. 2012; MAF 2011; Zhou et al. 2012) (Table 6). This comparison has been only undertaken with regards to the pesticide residues that have been detected/quantified in at least one sample in either the urban or the semi-rural area. Subsequently, using the LB approach, the estimated daily exposure levels to DDT, Endosulfan, Lindane, Chlorpyrifos methyl, Dimethoate, Malathion, Ethion, Procymidone, and Diazinon were within the range of estimates reported by studies carried out in other countries, while the exposure to Chlorpyrifos, Propoxur, Kresoxim- methyl as well as Dieldrin (semi-rural diet) was higher in Lebanon than other countries’ estimates. The opposite applies to the Fenitrothion, Primiphos methyl, and Fludioxinil estimates, which were, in Lebanon, lower than those reported by other studies.

**Table 6:** Mean estimated dietary exposure levels in Lebanon (μg/kg bw/day) as compared to mean intake levels reported by other total diet studies conducted in other countries at around the same period of time

	Lebanon (2009–2010) Adults, 25–55 years μg/kg bw/day				France (2007–2009) Adults, 18–79 years μg/kg bw/day		Cameroon (2006) Adult equivalent μg/kg bw/day		New Zealand (2009)^b^ Adult >25 years μg/kg bw/day	China (2007)^b^ Standard Chinese men: 18–45 years μg/kg bw/day
	Urban		Semi-rural							
Pesticide residue	LB	UB	LB	UB	LB	UB	LB	UB	LB	LB
DDT^c^	0	0.1	0.0035	0.13	ND	ND	ND	ND	M: 0.0099; F: 0.0073	0.016
Dieldrin	0.0000	0.10	0.0056	0.13	ND	ND	ND	ND	M:0.00004; F:0.00005	ND
Endosulfan	0.0080	0.39	0.0170	0.75	0.001	0.415	0.011	0.105	M: 0.0031; F: 0.0036	NI
Gamma HCH (lindane)	0.000	0.10	0.0008	0.12	0.001	0.176	ND	ND	ND	0.002^d^
Chlorpyrifos	0.0460	0.14	0.1261	0.24	0.013	0.141	ND	ND	M: 0.0023; F: 0.0022	NI
Chlorpyrifos methyl	0.0067	0.11	0.0004	0.12	0.005	0.135	ND	ND	M:0.0064; F:0.0062	NI
Fenitrothion	0.0000	0.10	0.0004	0.12	0	0.139	NI	NI	M:0.0132; F:0.0122	NI
Dimethoate	0.0431	0.14	0.0024	0.13	0.018	1.239	ND	ND	M: 0.02; F: 0.024	NI
Pirimiphos methyl	0.0109	0.11	0.0132	0.13	0.071	0.209	0.031	0.121	M: 0.106; F: 0.094	NI
Malathion	0.0000	0.10	0.0004	0.12	0	0.203	0.008	0.169	M:0.0025; F:0.0025	NI
Ethion,	0.0000	0.10	0.0018	0.13	0.001	0.117	NI	NI	M:0.00018; F: 0.00018	NI
Procymidone	0.0319	0.13	0. 0034	0.13	0.025	0.181	NI	NI	M: 0.0075; F: 0.0089	NI
Propoxur	0.0004	0.10	0.0016	0.12	ND	ND	NI	NI	ND	NI
Kresoxim-methyl	0.0066	0.11	0.0251	0.15	0	0.134	NI	NI	ND	NI
Fludioxonil	0.0018	0.10	0	0.12	0.065	0.229	NI	NI	M:0.04; F: 0.052	NI
Diazinon	0.0000	0.10	0.0010	0.12	0	0.133	ND	ND	M: 0.0005; F: 0.0005	NI

^a^Lebanon: present study

France; 2007–2009: (Nougadère et al. 2012)

Cameroon; 2006: (Gimou et al. 2008)

New Zealand; 2009: (MAF 2011)

China; 2007: (Zhou et al. 2012)

^b^If residue concentrations were less than the LOD, a value equal to zero was used in calculating dietary exposure estimates

^c^Only p,p’ DDD was detected in the Lebanese TDS while only 4,4’DDE was detected in the TDS of New Zealand

^d^Expressed as sum of α-HCH, β-HCH, γ-HCH, and δ-HCH

*M* males, *F* females

*ND* not detected

*NI* not investigated

The results of the UB approach are only compared to those reported by the TDSs conducted in France and Cameroon, (Nougadère et al. 2012; Gimou et al. 2008). Accordingly, estimated daily exposure levels for Lindane, Chlorpyrifos methyl, Fenitrothion, Malathion, Ethion, Procymidone, Kresoxim-methyl, and Diazinon were similar to estimates reported by France and Cameroon (when estimates were available). The estimated daily exposure levels for Dimethoate, Primiphos methyl, and Fludioxonil were, in Lebanon, lower than the estimates provided by the aforementioned studies (Nougadère et al. 2012; Gimou et al. 2008). On the other hand, the exposure to Dieldrin, DDT, Propoxur, Endosulfan, and Chlorpyrifos was higher in Lebanon than that reported by other studies, particularly in the semi-rural area (Nougadère et al. 2012; Gimou et al. 2008).

It is noteworthy that excluding animal-based products from the assessment undertaken in the present study may have largely underrepresented exposure to Organochlorine (OC), which are characterized by a very high solubility in fat and a tendency to accumulate in fatty animal tissues (Menard et al. 2008) (Ministry of Environment (Lebanon) et al. 2005; Hernandez et al. 1994). The consumption of animal-based foods is estimated at 355.4 g/person/day and 381.87 g/day in Beirut and Keserwan, respectively, thus representing, on a weight basis, 26.5 and 24.3 % of the urban and semi-rural diet (excluding beverages). The potential degree by which the intakes of OCs were underestimated in this study may be appraised when reviewing data published by other TDSs. For instance, the TDS conducted in China (Zhou et al. 2012) showed that 60 % of the daily intake of HCH was provided by meat and aquatic foods, against only 40 % by plant-based foods. Similarly, in the French TDS (Nougadère et al. 2012), 100 % of the intake of Lindane was provided by animal-based foods such as eggs, meat, poultry, and games. The same observations can be made for DDT and its metabolites, whereby the TDS conducted in China (Zhou et al. 2012) showed that 88.8 % of the total intake of DDT and its metabolites were provided by animal-based foods (meats, eggs, aquatic foods, and milk) against only 11.2 % by plant-based foods. The TDS conducted in New Zealand (MAF 2011) has also shown that exposure to total DDT residues was almost exclusively contributed by high-fat animal-based foods (chicken, eggs, fish, meat, dairy products). The fact that, in the present study, POPs were quantified in several samples, sometimes at levels exceeding the MRLs, raises concerns as to the population’s dietary exposure to the POPs in question and highlights the need, at the national level, for larger scale exposure studies that include animal-based foods, thus ensuring optimal coverage of food sources.

Concerning the food groups that were the major contributors (>5 %) to the dietary exposure to a given residue, the LB approach was adopted in the present study, since, with this approach, contribution levels do not depend on the LOD values for undetected results. For the post-harvest organophosphate insecticides, Primiphos-methyl and Chlorpyrifos-methyl, the main contributors were cereal-based products such as “breads,” “pasta and bulgur,” and “rice-based products.” For Chlorpyrifos, the most frequently detected residue in this study, the major contributors to intake included “cooked green leafy vegetables” and “pome and stone fruits.” One of the major contributors to Dimethoate included pome and stone fruits, followed by breads in the urban setting and “fruit salads” in the semi-rural diet. Cooked green leafy vegetables were a major contributor to the intake of Propoxur, followed by fruit salads. The main contributors to Procymidone exposure were “salads and raw vegetables” as well as “fruit-bearing vegetables,” while the main contributors to Kresoxim methyl exposure were “cooked green and leafy vegetables” in both the urban and rural diets. Only one contributor was identified for Diazinon (tropical fruits,” semi-rural), Ethion (fruit juices, semi-rural), Fenitrothion (stem vegetables; semi-rural), Malathion (“Brassica vegetables and artichoke,” semi-rural), and Fludioxonil (fruit salads, urban). For the POP Dieldrin, which was detected/quantified in some food samples from the semi-rural area, “tropical fruits” were the major contributor to its intake followed by “cooked green leafy vegetables.” The major contributors to the other organochlorine pesticide residues were “grapes and strawberries” (alpha and beta endosulfan), “salads and raw vegetables” (endosulfan sulfate) in addition to pasta and bulgur (DDD,pp. and lindane).

The results of the present study should be interpreted in light of the following limitations. First, the study was limited to two areas in Lebanon: Greater Beirut (urban) and Kesrwan (semi-rural). Beirut was chosen because it comprises 40 % of the Lebanese population and is usually considered as the melting pot of the country. The inclusion of the semi-rural area may be considered as a pilot study allowing the preliminary investigation of potential differences in dietary exposure between regions. Furthermore, this study was confined to the adult fraction of the population similarly to several other exposure assessments conducted around the world (Gimou et al. 2008; Zhou et al. 2012).

However, the selection of the adult participants for this study has led to the exclusion of infants and young children, who may be more vulnerable to the potential health effects of pesticide residues. In fact, the child’s developmental processes and mechanisms may be disturbed by exposure to pesticide residues (Hulin et al. 2014), and the metabolic pathways of detoxification may not be as efficient in children as in adults. In addition, young children may be exposed to higher concentrations of pesticide residues through their diet because the ratio of the quantity consumed to body weight is higher in this population group than in adults (Hulin et al. 2014). It is therefore crucial that future TDSs focus on these vulnerable population groups, when assessing dietary exposure to pesticide residues. The present study was also restricted to plant-based foods, which has undoubtedly resulted in a suboptimal coverage of OCs, but should have allowed a relatively adequate exposure assessment and risk characterization for the other pesticide residues under investigation, given that plant-based products including fruits, vegetables, and cereals represent the main intake source of these residues (Menard et al. 2008). The vegetarian population may be exposed to some pesticide residues more than the general population due to their higher consumption of fruits, vegetables, and cereals (Van Audenhaege et al. 2009). However, the present study did not evaluate the dietary exposure of this specific population subgroup to pesticide residues. It is worth mentioning that acute exposure and acute risk cannot be assessed within the protocol of this study, which focuses on “background levels” and chronic dietary exposure. In fact, the deterministic approach adopted in this study, coupled with the composite sampling of food items, prevents the assessment of probabilities that a consumer’s exposure, on a given day, might exceed an acute reference value (EFSA 2012). Several of the detected pesticides in this study were organophosphate insecticides, which may pose acute toxicity concerns (Fenske et al. 2002). It is therefore possible that exposure to organophosphates, based upon the distribution of residues in individual food items (not composites) and a high consumption rate of these food items on a single day, could result in higher exposure percentile (i.e., 90, 95, and 99 %) that may exceed the ARfD. Finally, acknowledging that individual members of the organophosphate insecticide family possess similar toxicological mechanisms of action, it is recommended that future studies assess the consumer’s dietary exposure to the entire family of organophosphates rather than to only individual chemicals (Boobis et al. 2008).

## Conclusion

This paper responds to the need for data on dietary exposure to pesticide residues in Lebanon and the characterization of the consumer’s risk. While not excluding the possibility that the daily exposure levels determined in the present study may not be representative of the Lebanese population as a whole, this study has provided a first estimate of the consumer’s dietary exposure to pesticide residues in Lebanon. These estimates were generated by using the TDS protocol, and by using two approaches in estimating dietary exposure, the LB approach, which generally underestimates contamination and exposure levels and the UB approach, which tends to overestimate these values (EFSA 2010). Only few studies have applied these approaches in their dietary exposure assessment (Nougadère et al. 2012; Gimou et al. 2008). According to the study’s findings, exposure levels for most of the investigated pesticides are within the range of those reported by other countries while being far from the respective ADIs. However, the study has shown that, with the UB approach, exposure levels to Dieldrin and Diazinon may be high, particularly in the semi-rural area. This finding, coupled with the detection/quantification of several other OCs in the analyzed food samples stresses the necessity of future exposure assessment studies focusing on animal-based foods and exposure to OCs in Lebanon. The findings of this study call for (1) undertaking larger-scale national studies with a more comprehensive scope in exposure assessment and a broader representation of the country’s multiple regions, (2) building capacities with regards to analytical techniques to widen the scope of investigations of pesticide residues in foods and (3) setting up robust surveillance systems that monitor both the levels of pesticide residues in foods and the use of illegal pesticides.
